# Investigation of Off-Season Breeding Effects on Egg-Laying Performance, Serum Biochemical Parameters, and Reproductive Hormones in Zhedong White

**DOI:** 10.3390/vetsci12020179

**Published:** 2025-02-17

**Authors:** Jiaqiao Zhu, Yonggang Ma, Waseem Ali, Rui Yu, Hui Zou, Zongping Liu

**Affiliations:** 1 College of Veterinary Medicine, Yangzhou University, Yangzhou 225009, China; jqzhu@yzu.edu.cn (J.Z.); 007854@yzu.edu.cn (Y.M.); 008301@yzu.edu.cn (W.A.); 1759006631@163.com (R.Y.); zouhui@yzu.edu.cn (H.Z.); 2 Jiangsu Co-Innovation Center for Prevention and Control of Important Animal Infectious Diseases and Zoonoses, Yangzhou 225009, China; 3 Joint International Research Laboratory of Agriculture and Agri-Product Safety of the Ministry of Education of China, Yangzhou University, Yangzhou 225009, China

**Keywords:** Zhedong white goose, off-season reproduction, seasonal reproduction, egg-laying performance

## Abstract

Off-season breeding is a method of encouraging animals through manual intervention to reproduce during the non-breeding season. The egg-laying period of the Zhedong white goose usually starts from September and ends in April of the following year. During the summer months from May to August, with long daylight hours and high temperatures, the white geese rest and do not lay eggs. This study explored the effects of off-season breeding on egg production performance, serum biochemical indicators, and hormone secretion in Zhedong white goose. The experiment was divided into normal breeding and off-seasonal breeding. The results suggested that the peak laying period lasted 3 months, and egg production was 15.62% higher than that in the control group in the off-season breeding group. The off-season breeding group had a 4.13% higher egg fertilization rate in November (laying period) than the control group, and the hatching rate of hatched eggs and fertilized eggs in the peak laying period was 2.77% and 2.3% higher, respectively, than the control group. The experimental group showed significantly higher serum P, ALB (albumin), TG (triglyceride), and TC (total cholesterol) levels; serum AST (glutamic oxalacetic transaminase) activity; and CREA (creatinine) and BUN (blood urea nitrogen) levels than the control group at different time points. The serum LH (luteinizing hormone), FSH (follicle-stimulating hormone), P4 (progesterone), and E2 (estradiol) levels were significantly higher in the off-season breeding group than in the control group. The serum T3 (triiodothyronine) and T4 (Tetraiodothyronine) levels were significantly higher in the off-season breeding group than in the control group at different points in the breeding cycle. The control group showed a significantly higher gene expression of GnRH (gonadotropin releasing hormone) in the hypothalamus; GnRH, FSH, and LH in the pituitary; and GnRH in the ovary and significantly lower gene expression of VIP (vasoactine intestinal peptide) and PRL (prolactin) in the pituitary than the off-season breeding group. Thus, the off-season breeding of Zhedong white geese may prolong the peak laying period and improve egg production performance, thus enhancing the economic benefits of goose breeding.

## Simple Summary

This study investigates the effects of off-season breeding on Zhedong white geese egg production performance, physiological functions, and reproductive hormones. In this study, off-season breeding significantly changed Zhedong white geese serum biochemical indicators and some key hormones, which effectively increased egg production. These results provide a theoretical basis for the application of this breeding strategy in Zhedong white goose.

## 1. Introduction

China has the highest number of geese worldwide. In 2021, the number of commercially bred geese in China was approximately 570 million, accounting for more than 90% of the global production [1]. Xiangshan has become the largest breeding base of Zhedong white geese in China. The county has a total of 1.26 million geese in stock and produces and sells 10 million goslings annually. The total output value of the entire industrial chain has reached CNY 560 million. Zhedong white goose, also known as Xiangshan white goose and Zhejiang white goose, is a medium-sized goose bred for their meat and liver. Zhedong white geese are typically seasonal breeders; the breeding cycle begins at the end of autumn as the days gradually shorten (from the end of September to the beginning of October) and ends at the end of spring (from April to June) as the days gradually lengthen, and then enters the rest period in summer [2]. The average reproductive cycle of the Zhedong goose is 150–180 days, which includes three to four laying periods; 8–11 eggs are laid in each laying period, the duration of each laying period is 15–40 min, and the average laying interval is 24–40 h. Thus, typically, 30–45 eggs are produced by a female goose annually; the average egg weight is 160–170 g, and the fertilization rate is 85% [3]. Therefore, how to effectively prolong the breeding cycle of Zhedong white geese is very important.

Off-season breeding aims to use human intervention to make geese produce eggs in the non-breeding season contrary to their physiological characteristics [4]. Previous studies have successfully used light regulation and forced molting to induce geese to produce eggs in the summer of the second year and achieved high-temperature egg production in the summer of the third year [5]. Although the average number of eggs produced in the off-season breeding cycle was lower than that in the normal breeding cycle, the price of goslings produced in the summer was much higher than the price of goslings raised in the normal breeding cycle, and the production cost was divided between the two seasons. The implementation of feeding technology in Landes geese was reported to increase the average profit by 20% compared with that generated using the normal breeding cycle, which affirms the potential of developing off-season breeding of geese in China. With the economic development and the consequent increase in food consumption, the market demand for goose products is gradually growing. The application of off-season breeding can effectively induce Zhedong white geese to produce eggs in the non-breeding season, which has economic benefits and ensures a stable supply to the market.

It is not difficult to find that counter-seasonal breeding can effectively increase economic benefits. It is widely acknowledged that animals, particularly birds, mainly regulate the seasonality of reproduction and other physiological activities by depending on the annual variations of the daily light cycle [6]. Egg production requires the synthesis of large amounts of lipids, proteins, and minerals. When laying starts, plasma triglycerides increase substantially and lipoproteins are needed to transport them from liver to ovary [7]. In a previous study, the elevation of TG and ALB indicated that the lipid and protein synthesis capacity of liver is greatly improved during egg laying [8]. The study also found that egg production requires large amounts of trace elements, especially Ca and P, to form eggshell, and the transport of Ca requires participation of Na and Cl [9]. It is well known that hormonal changes are closely related to the reproductive performance of animals. Bédécarrats et al. found that the photoperiod signals by the pineal gland, acting on photoreceptors located in the retina and the deep brain, directly regulate GnRH, and indirectly affect GnIH secretions through melatonin secretions [10]. GnRH and GnIH, respectively, stimulate and inhibit secretions of luteinizing hormone (LH) and follicle-stimulating hormone (FSH) through the pituitary gland so as to activate or inhibit reproductive activities. In addition, photo-signals trigger the release of thyroid-stimulating hormone (TSH) by stimulating the photoreceptor cells. TSH stimulates the local conversion of thyroid hormone total thyroxin (T4) into triiodothyronine (T3), which may in turn stimulate the release of GnRH-I in the median eminence, which subsequently induces reproductive activation [11]. Therefore, hormone regulation plays an essential role in the application of off-season breeding. A growing body of evidence suggests that serum biomarkers have been developed as indicators for clinical and breeding purposes in humans and animals [12,13]. In animal breeding, serum parameters serve as indirect indicators for both animal health and economic traits [14]. Such as high levels of creatine kinase (CK) and lactate dehydrogenase (LDH) are associated with pale, soft, exudative meat. Qi et al. found that total protein (TP), albumin (ALB), globulins (GLB), total cholesterol (CHOL), total triglyceride (TG) content, and glutamic-pyruvic transaminase (GPT) are related to egg production [13].

There is no doubt that the application of off-season breeding can effectively induce Zhedong white geese to produce eggs in the non-breeding season, which has economic benefits and ensures a stable supply to the market. However, because off-season breeding is a production strategy that goes against physiological laws, it remains unknown whether it affects the egg laying performance, physiological functions, and reproductive endocrine regulation of Zhedong white goose. Therefore, this study aims to investigate the effects of off-season breeding on the egg-laying performance, physiological functions, and reproductive hormones of Zhedong white geese.

## 2. Materials and Methods

### 2.1. Experimental Design

This study was conducted at an eastern Zhejiang white goose breeding farm located in Xiangshan County, Ningbo City, Zhejiang Province, China. Experiments were approved by the Animal Ethics Committee of the university (Yangzhou, China; approval no. SYXK (Su) 2022-0044). A total of 2000, 120 days, female Zhedong white geese of the same batch were selected; the number of male geese was 4000, 120 days. An amount of 1000 each were allocated to the normal seasonal breeding (control) group and off-season breeding group, respectively. Male geese were housed with female geese in a ratio of 2:1 to facilitate mating in each group. In the control group, geese were housed in a facility equipped with automatic feeders and egg laying houses furnished with appropriate bedding materials, and they were provided with ample space for activity along with an artificial pool outdoors. In contrast, geese in the off-season breeding group were accommodated in a light-protected breeding shed with controlled lighting conditions. The method was as follows: 1. Lighting Control. Implement artificial lighting: from 6 a.m. to 6 p.m., provide artificial supplementary lighting to the goose house for 10 to 12 h each day, which can increase the duration of light in the goose house to over 16 h; Adjust the intensity of light: farmers can control the number and intensity of external light sources to adjust the intensity of light. Generally, an intensity of 1500 to 2000 lux is appropriate. 2. Environmental Regulation. 1. Adjust the temperature in the goose house: a room temperature of 15 to 20 degrees Celsius is suitable for the egg-laying and reproduction of geese. During off-season reproduction in winter, heaters or radiators can be used to maintain the temperature in the goose house, allowing geese to live in a suitable temperature to increase the reproduction rate. 2. Maintain the humidity in the goose house: appropriate humidity can help geese maintain their body’s water balance and normal metabolism. During off-season reproduction in winter, the humidity in the goose house should be maintained at 60% to 70%. Spraying water or adding humidity equipment can achieve this goal.

### 2.2. Sample Selection

A total of 100 Zhejiang white geese were randomly selected from the off-seasonal breeding group and the control group for blood collection in 7, 8, 9 months. Fasting was ensured for 12 h before blood collection. In total, 5 mL of whole blood was collected from the wing vein using vacuum blood collection vessels, and the whole blood samples were kept at 4 °C for 30–60 min. The whole blood was centrifuged in a refrigerated centrifuge for 10 min, after which, the supernatant was drawn in a 5 mL centrifuge tube and stored at −20 °C.

### 2.3. Experimental Period

The control group (normal seasonal breeding) underwent an experimental period spanning 11 months, from 1 August 2018 to 1 June 2019. This period encompassed both the rest phase (August to September 2018 and May to June 2019) and the egg laying phase (October 2018 to March 2019), which included the peak egg laying periods in December 2018 and January and March 2019.

For off-season breeding group, the experimental period lasted a total of 11 months—from 1 April 2018 to 1 February 2019—including the body production period (April to May 2018, December 2018, and January and February 2019), egg laying period (June to November 2018), and peak egg laying period (August to October 2018).

### 2.4. Implementation of Off-Season Breeding Strategy

Throughout the entire experimental period, the control group was afforded opportunities for physical activity and provided with ample water and feed in accordance with the standard seasonal breeding protocol to uphold a regular laying cycle and rest period. In August 2018, the control group entered the laying phase. From 1 August to 1 June 2019, eggs were collected every morning and evening, and the total egg number and number of fertilized eggs, hatched goslings, and lost young geese (dead-in-shell) were determined.

The feed intake in the off-season breeding group was restricted from 1 to 20 February 2018; the total intake of fine and coarse feed was maintained at 150 g per animal, and green feed (spartina anglica) was provided ad libitum. From 1 February, the geese were driven into the shed every day after sunset and continued to be given artificial light, and the long light treatment was maintained for 18 h a day. During this period, the nutritional status of the breeding geese in the off-season breeding group was poor, so they gradually stopped egg production. From 21 February to 1 March, the feed intake for geese in the off-season breeding group was gradually increased to 200–250 g per animal, and the light exposure period was maintained at 18 h per day. During this period, the geese in this group began to molt. From 1 March to 31 March, normal feeding was resumed, with 200–250 g feed concentrate per animal, and green feed was provided ad libitum. From March 1, the geese in the off-season breeding group shed their feathers. From 1 to 30 April, 200–250 g feed concentrate was administered per animal, and light exposure was increased by 1 h every week from 15 April to 1 June. After 1 month of molt recovery, feathers grew back and egg production started. From June 1, a shorter light exposure period of 12 h a day was used to promote egg production; the temperature of the goose house was maintained below 25 °C. Other management methods and feeding materials were the same as those in the control group, and each house was swept once to ensure clean conditions. From 1 April 2018 to 1 February 2019, eggs were collected once every morning and evening and screened to identify fertilized eggs. The total egg number and numbers of fertilized eggs, goslings born, and young geese lost in the 11-month production cycle were determined. Table 1 shows the composition of the feed provided to Zhedong white geese during the laying period.

The nesting behavior of geese in the control and off-season breeding groups was checked once every 3–4 d. If nesting behavior was observed, the feed intake of the geese was restricted for 7–10 d to promote the termination of nesting, and the birds were returned to the flock.

### 2.5. Detection of Serum Biochemical Indicators

By employing an automatic blood biochemical analyzer, the indices related to protein metabolism, fat metabolism, mineral metabolism, liver function, and kidney function of geese were measured. The determined indicators encompass the following: protein metabolism indicators such as total protein (TP), albumin (ALB), and globulin (GLOB); mineral metabolism indicators like calcium (Ca) and phosphorus (P); fat metabolism indicators including total cholesterol (TC) and triglycerides (TG); liver function indicators, namely aspartate aminotransferase (AST), alanine aminotransferase (ALT), total bilirubin (TBU), gamma-glutamyl transferase (GGT), creatine kinase (CK), and lactate dehydrogenase (LDH); and kidney function indicators, creatinine (CREA) and blood urea nitrogen (BUN).

### 2.6. Examination of the Content of Serum Hormone E2, P4, FSH, LH, PRL, and T4

After serum sample preparation, ELISA was performed according to the kit protocol. Finally, with the optical density (OD) value of the measured standard as the abscissa and the concentration value of the standard as the ordinate, a standard curve was drawn using Excel, and a linear regression equation was obtained. The OD value of the sample was substituted into the equation to calculate the concentration of the sample.

### 2.7. Examination of the Gene Expression of Hypothalamus, Ovary Tissues FSH, LH, PRL, GnRH, and VIP

According to the mRNA sequences of actin, FSH, LH, PRL, gonadotropin-releasing hormone (GnRH), and VIP of geese in GenBank, qRT-PCR primers were designed by Oligo 7.6 software, and the primer sequences were finally obtained by NCBI Primer Blast for comparison. All primers were synthesized by BGI Ltd. (Shenzheng, China). The primer sequences of target genes are listed in Table 2.

The cDNA template was processed in steps according to the reference instructions provided by the kit. After qRT-PCR, the tissue specificity of the primers was determined according to the dissolution curve. β-actin was used as the internal reference, and the gene expression was calculated using the 2^−ΔΔCt^ method.

### 2.8. Data Statistics and Analysis

All data are expressed as the as mean ± SEM. Statistical analyses were performed using GraphPad Prism version 8.02. and the differences between the two groups were analyzed using the *t*-test. Statistical significance was declared. “*” denotes a significant difference compared with the control group (*p* < 0.05); “ns” indicates no significant difference compared with the control group (*p* > 0.05).

## 3. Results

### 3.1. Effect of Breeding Season on the Reproductive Performance of Eastern Zhejiang White Geese

In this study, the reproductive performance during the whole breeding cycle (11 months) of the off-season breeding and control group of eastern Zhejiang white geese was examined (Table 3). The hatching rate of eggs in the off-season breeding group was 1.58% higher than that in the control group, and the hatching rate (numbers of hatching geese/number of eggs) was 0.95% higher than that in the control group. The average economic benefit per goose was 35.5 RMB higher in the off-season breeding group than in the control group in the breeding cycle from April 2018 to June 2019.

### 3.2. Effect of Breeding Season on Production Performance of Zhedong White Geese

**Figure 1:**
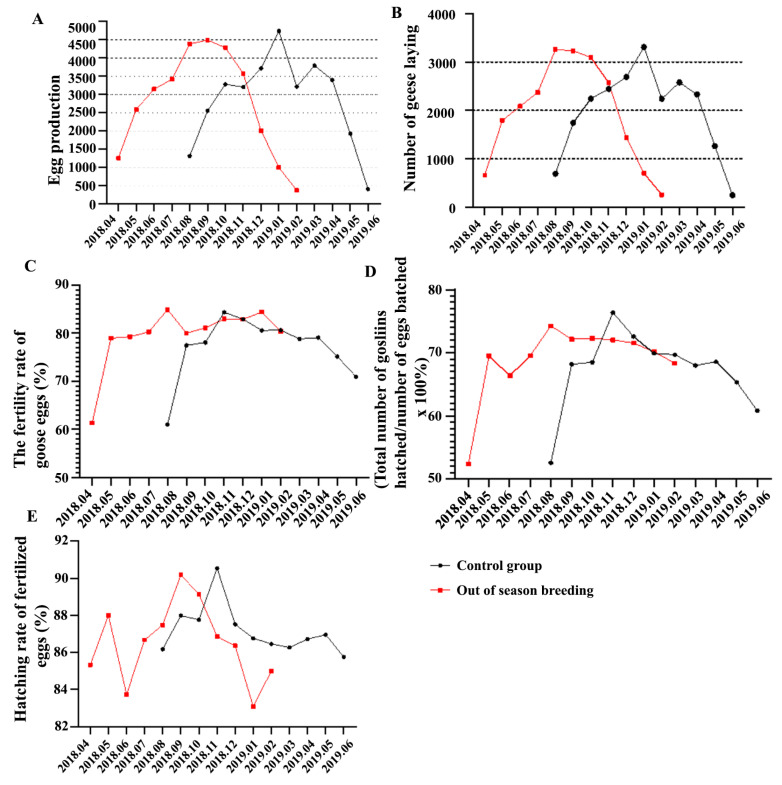
Effect of off-season breeding on the production performance of eastern Zhejiang white geese. (**A**) Egg production of Zhedong white geese; (**B**) Nestlings of Zhedong white geese; (**C**) Egg fertilization rate of Zhedong white goose; (**D**) Hatching rate of Zhedong white geese; (**E**) The hatchability of fertilized eggs of Zhedong white geese.

As shown in Figure 1A, the off-season breeding group maintained the peak period of laying (more than 4000 eggs per month) for three consecutive months (August to October) during the laying period, whereas the control group exhibited peak laying only in January. The egg production in the off-season breeding group was 15.62% higher than that in the control group. In the off-season breeding group, egg production decreased in December and entered the perinatal period, which lasted for 6 months. As shown in Figure 1B, the number of chicks in the control group reached more than 3000 only in January during the peak laying period, whereas the number of chicks in the off-season breeding group reached more than 3000 for three consecutive months during the peak laying period, which was 20.60% higher than that in the control group. As shown in Figure 1C, the fertilization rate of the off-season breeding group was 1.58% higher than that of the control group. In November, the egg fertilization rate in the off-season breeding group was 4.13% higher than that in the control group. The egg fertilization rate in the off-season breeding group in December, January, and February was 3.81%, 9.3%, and 9.57% higher, respectively, than that in the control group. As shown in Figure 1D, the hatching rate of the control group was 0.95% lower than that in the off-season group during the whole breeding cycle. The egg hatching rate during the peak laying period and rest period (January to February) in the off-season breeding group was 2.77% and 6.24% higher, respectively, than that in the control group. As shown in Figure 1E, there was no significant difference in the hatching rate of fertilized eggs between the two groups during the whole breeding period, but the hatching rate of fertilized eggs in the off-season breeding group was 2.3% higher than that in the control group at the peak of laying.

### 3.3. Effects of Breeding Season on Serum Biochemical Indexes of Zhedong White Geese

**Figure 2:**
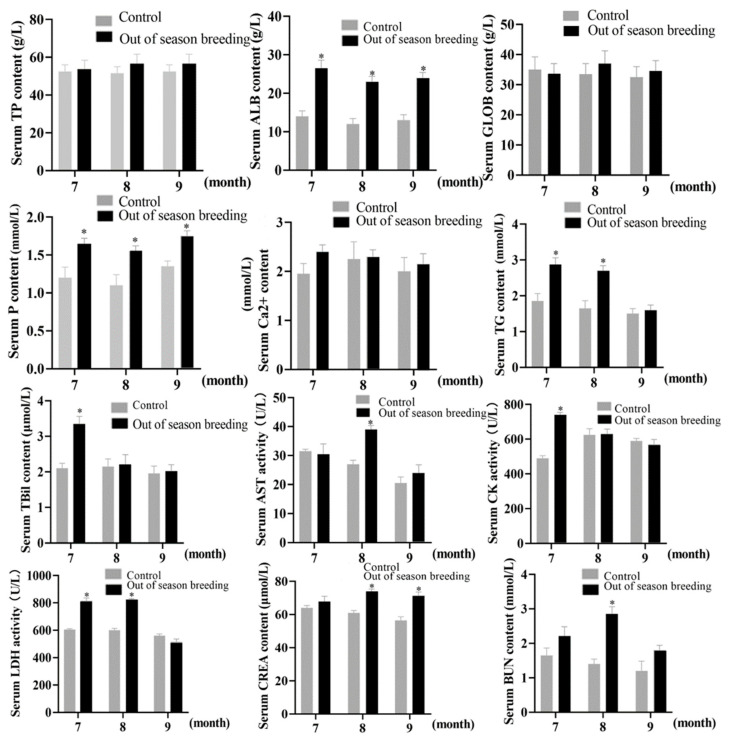
Effects of off-season breeding on serum biochemical indexes of Zhedong white geese. ELISA kit was used to analyze the serum TP, ALB, GLOB, P, Ca^2+^, TG, TBil, AST, CK, LDH, CREA, and BUN content. * *p* < 0.05 indicates a significant difference in comparison with the control.

As shown in Figure 2, compared with the control group, there was no significant difference in serum TP and GLOB levels in the control and off-season breeding groups (*p* > 0.05), and serum ALB content was significantly higher in the off-season breeding group than in the control group (*p* < 0.05 or *p* < 0.01). Next, we examined the effect of off-season breeding on serum ion levels in eastern Zhejiang white geese. The results showed that the serum P content of the off-season breeding group was significantly higher than that of the control group (*p* < 0.05), but the serum Ca content was not significantly different (*p* > 0.05). The results of serum lipid metabolism showed that the serum triglyceride (TG) levels in the off-season breeding group were significantly higher than those in the control group (*p* < 0.05). Analysis of serum liver function showed that the serum TBil content in the off-season breeding group was significantly higher than that in the control group in July (*p* < 0.05). The activity of CK, LDH, and AST in the serum were significantly different from that in the control group at various time points (*p* < 0.05). The serum CREA level of the off-season breeding group was significantly higher than that of the control group in August and September (*p* < 0.05), and the serum BUN content was extremely significantly higher than that of the control group in August (*p* < 0.01). There was no significant difference at other time points (*p* > 0.05). The above results showed that the off-season breeding of eastern Zhejiang white geese increased the serum ALB, TG, and P levels and AST activity, promoted the metabolism of proteins, fats, and minerals, and helped to maintain egg production performance at the peak of laying and showed that off-season breeding had no adverse effects on serum biochemical indexes of eastern Zhejiang white geese.

### 3.4. Effects of Breeding Season on Serum Levels of Reproductive Hormones in Zhedong White Geese

**Figure 3:**
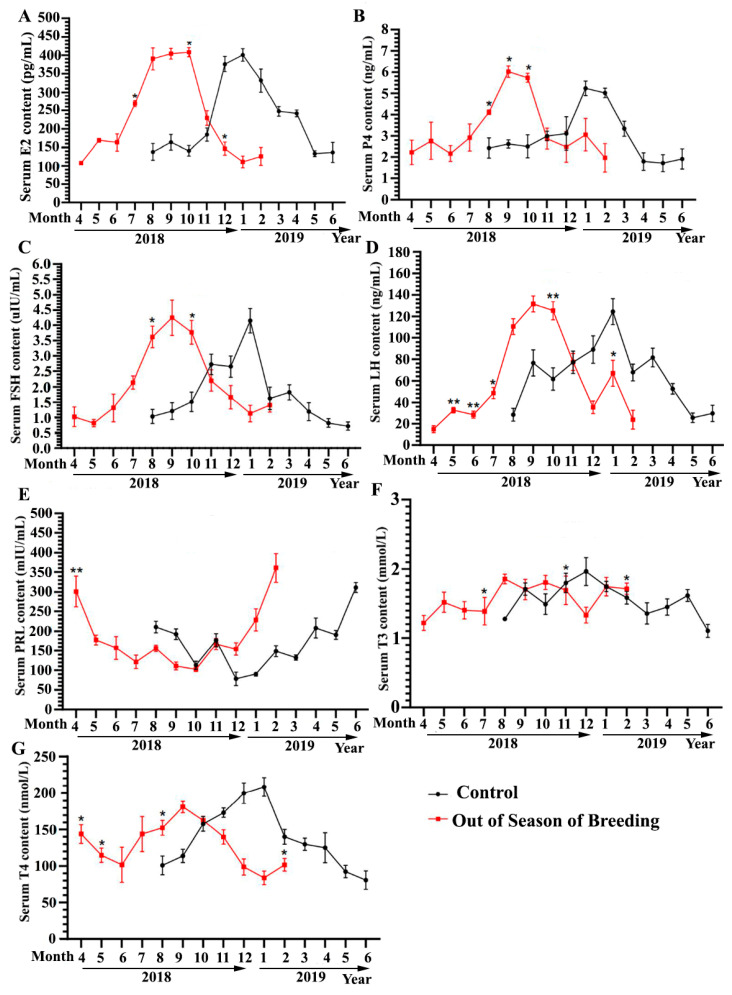
Effects of off-season breeding on serum reproductive hormone levels of Zhedong white geese. (**A**–**G**) ELISA kit was used to analyze the serum E2, P4, FSH, LH, PRL, T3, and T4 content; * *p* < 0.05 indicates a significant difference, and ** *p* < 0.01 indicates a very significant difference.

As shown in Figure 3A,B, the changes in serum E2 and P4 levels in the two groups were basically the same. The serum E2 content in the off-season breeding group was significantly higher than that in the control group at the corresponding time points in April and July (*p* < 0.05). The serum E2 content in the off-season breeding group was significantly lower than that in the control group at the 12th month (*p* < 0.05), and there was no significant difference between the two groups at other time points (*p* > 0.05). The serum P4 content in the off-season breeding group was significantly higher than that in the control group at corresponding time points from May to July (*p* < 0.05), and there was no significant difference between the two groups at other time points (*p* > 0.05).

As shown in Figure 3C,D, the trend of change in serum FSH and LH levels was similar between the two groups, and the highest levels of these hormones were consistent with the peak egg laying period. The serum FSH content in the off-season breeding group was significantly higher than that in the control group at the corresponding time points in August and October (*p* < 0.05), and there was no significant difference between the two groups at the other time points (*p* > 0.05). The serum LH content of the off-season breeding group was significantly or extremely significantly lower than that of the control group at corresponding time points from February to April (*p* < 0.05 or *p* < 0.01), whereas the serum LH content of the off-season breeding group was significantly higher than that of the control group at the 7th and 10th months (*p* < 0.05); there was no significant difference between the two groups at other time points (*p* > 0.05).

As shown in Figure 3E, the trend of change in serum PRL content was similar in the two groups, and the highest hormone level was consistent with the rest period. The serum PRL content of the off-season breeding group was significantly higher than that of the control group at the corresponding time points in the first month after the start of breeding (*p* < 0.05), and there was no significant difference between the two groups at the other time points (*p* > 0.05). PRL promotes the reproductive behavior of some animals and enhances the maternal nature of female animals, especially clinginess in birds.

As shown in Figure 3F,G, the changes in serum T3 and T4 levels were consistent between the two groups. The serum T3 levels in the two groups fluctuated, and the overall level was higher at the peak of the laying period. The trend of change in serum T4 content in the two groups was consistent with that in the peak laying period, with a lower level in the rest period and a higher level at the peak of laying. The serum T3 content in the off-season breeding group was significantly higher than that of the control group at the corresponding time points in the 8th and 11th month (*p* < 0.05), and was significantly lower than that of the control group in the 4th month (*p* < 0.05). There was no significant difference between the two groups at other time points (*p* > 0.05). The serum T4 content in the off-season breeding group was significantly higher than that in the control group at the corresponding time points in January to February and November (*p* < 0.05), and was significantly lower than that of the control group in May (*p* < 0.05). There was no significant difference between the two groups at other time points (*p* > 0.05). The above results suggest that off-season breeding affected only the time and season of laying, and the change trend of the contents of reproduction-related regulatory hormones detected throughout the off-season breeding cycle was consistent with those in the normal cycle.

### 3.5. Effects of Breeding Season on the Expression of Hormone-Related Genes in Eastern Zhejiang White Geese

**Figure 4:**
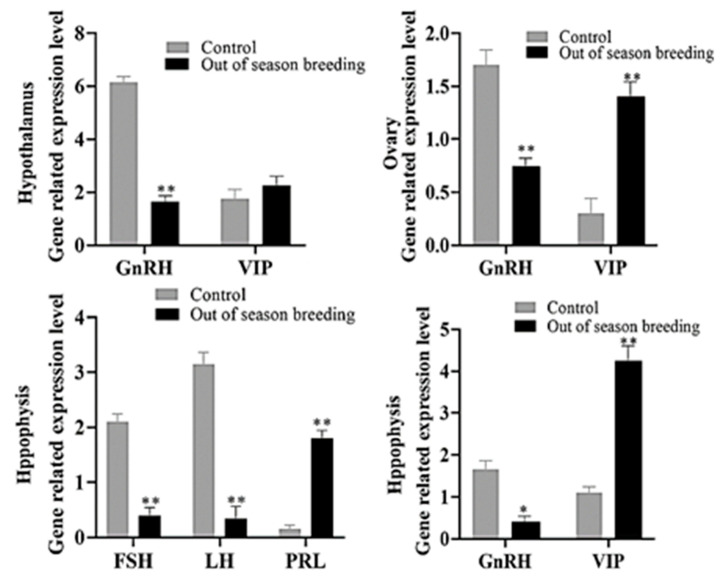
Effects of off-season breeding on the expression of reproductive hormone-related genes in Zhedong white geese. q-RT-PCR was used to analyze the expression of hormone-related genes. * *p* < 0.05 indicates a significant difference; ** *p* < 0.01 indicates a very significant difference.

In December, the tissues of hypothalamus and ovary of the two groups of Zhedong white geese were collected. At this time, the off-season breeding group began to enter the fallow period, and the control group was in the high-yield period. As shown in Figure 4, the expression of GnRH in the hypothalamus was significantly higher in the control group than in the off-season breeding group (*p* < 0.01), but there was no significant difference in the expression of the VIP gene between the two groups (*p* > 0.05). The expression levels of the FSH, GnRH, and LH genes in the control group were significantly higher than those in the off-season breeding group (*p* < 0.05 or *p* < 0.01), and the expression levels of the PRL and VIP genes in the control group were significantly lower than those in the off-season breeding group (*p* < 0.05 or *p* < 0.01). The expression of the GnRH gene in the control group was significantly higher than that in the off-season breeding group (*p* < 0.01), and the expression of the VIP gene in the control group was significantly lower than that in the off-season breeding group (*p* < 0.01).

## 4. Discussion

The technology of goose off-season reproduction is designed to carry out egg production in the non-reproductive season through artificial intervention against physiological characteristics. Natural light variation is the main factor for the emergence of seasonal reproductive trait characteristics in many animals. The implementation of goose out-of-season breeding production technology is a systematic engineering, the key of which is light and forced moult. Many studies have found that changing the light can affect the reproductive performance of poultry [15,16]. In this study, we successfully established an off-season breeding production technology for Zhedong white geese by limiting feed-forced molting combined with light and temperature regulation. We found that the egg laying peak of the off-season breeding group increased by 15.62% compared with the control group.

Serum biochemical indexes serve as vital parameters for evaluating the nutritional status, physiological function, and production performance of animals in commercial production [17]. The higher the serum content of TP and ALB, the better the absorption and deposition of the protein provided by the feed, the higher the feed utilization rate, and the higher the protein metabolism [18]. The present study found that there was no significant difference in serum TP concentration between the off-season breeding group and the control group at the peak of the laying period, but the serum ALB content was significantly higher than that of the control group, indicating that the off-season breeding of eastern Zhejiang white geese did not affect protein absorption and metabolism and facilitated protein deposition at the peak of the laying period, which is conducive to maintaining their egg production performance.

Serum biochemistry considered to be a good indicator of health status. Ca and P, the most abundant mineral elements in the body, are crucial for bone growth, muscle development, and the maintenance of egg production performance and physiological function homeostasis during the breeding period [19]. Micaela Sinalair-Black et al. found that serum Ca and P ion levels were correlated with egg laying performance, and blood calcium concentration was related to eggshell formation [20]. Evandro Ferreira Cardoso et al. found that when laying hens suffered from heat stress, their serum Ca and P levels decreased significantly [21]. Alagawany et al. found that different P levels in feed materials had no significant impact on the TP, AST, ALT, and BUN levels [22]. In the present study, it was found that the serum P and Ca levels at the peak of egg production were significantly higher in the off-season breeding group than in the control group, but there was no significant difference, indicating that the off-season breeding of eastern Zhejiang white geese did not affect the absorption and metabolism of mineral Ca and P, and the off-season breeding group increased the utilization efficiency of Ca and P in the feed during egg production, which was conducive to maintaining a high-yield performance. In short, Ca and P content is crucial for maintaining egg production performance.

Hen egg yolk is a natural supramolecular assembly of lipids and proteins with different organization levels [23]. The determination of serum lipid metabolism indexes is crucial for evaluating lipid metabolism. The liver synthesizes majority of the body’s cholesterol, while exogenous cholesterol obtained from dietary intake is absorbed through the intestinal tract. Kuzmenko et al. found that serum TG levels fluctuate with season, typically being higher in summer than in winter [24]. This is one of the reasons why poultry produce fewer eggs in winter. Lipids such as TG and cholesterol in the body enter the oocytes through specific receptors under the regulation of reproductive hormones, which promotes the formation of egg yolk. Maintaining normal levels of serum TG and TC provides nutritional guarantee and improves the hatching rate of fertilized eggs [25]. Mourot et al. [26] found that the likelihood of fatty liver was twice as high in Landes geese than in Polish geese, and the serum TG levels in Landes raised for foie gras (foie gras of animals, especially geese, usually eaten as foie gras) were higher and they exhibit higher fat metabolism and deposition in the liver. Tanabe et al. reported that changes in serum TG levels were significantly positively correlated with laying performance in laying hens [27]. When the serum TG levels decreased, the laying performance decreased, and when the serum TG levels recovered and increased, the laying performance gradually increased. In the present study, it was found that the serum TG levels in the off-season breeding group were significantly higher than those of the control group at the peak of the laying period, and the serum TC content also showed an increasing trend, indicating that the off-season breeding of Zhedong white geese did not affect the absorption and metabolism of lipids in the feed, and the higher serum TG and TC levels would help to maintain the energy demand and the nutritional composition of egg yolk at the peak of the laying period.

The concentrations of serum AST and ALT are related to the metabolism and function of the liver in farm animals. After liver cell damage, the concentrations of aspartate aminotransferase and alanine aminotransferase in the blood are usually higher [28]. The liver plays an important role in lipid metabolism, promoting the absorption of fat-soluble vitamins and synthesis and the decomposition of proteins, regulating blood glucose, and participating in hormonal regulation. Jiang Tao [29] reported that seasons significantly affect the liver indexes in different goose breeds; for example, the serum AST activity is higher in summer than in winter, which is more conducive to protein absorption. In the present study, the serum AST activity of the off-season breeding group was slightly higher than that of the control group at the peak of egg production, and significantly higher than that of the control group in August, which was consistent with the study results of Jiang Tao. In conclusion, feeding Zhedong white geese in off-season not only increased egg production but also promoted the increase in serum AST activity during the laying peak, thus improving protein deposition and maintaining high egg-production performance. Kang Bo et al. conducted blood biochemical analysis in white geese and seed geese in northeast China and found significant differences in ALT, AST, and GGT activity and other indicators but no significant differences in TBil and Dbil between the goose breeds [30]. Previous studies indicate that increasing the proportion of protein in the feed of lion head geese increased the egg production and hatching rate, serum ALT activity, and expression of pituitary FSHP and ovarian FSH receptor, which improved the production performance of lion head geese [31,32]. In the present study, there was no significant difference in serum Tbil levels and ALT and GGT activity between the experimental and control groups, indicating that off-season breeding did not affect the liver function of eastern Zhejiang white geese.

It is well known that poultry hormone regulation plays an important role in reproduction, which is conducive to maintaining the physiological pressure of poultry and improving economic benefits. Birds are mostly seasonal and their reproductive process is initiated by environmental queues [33]. In poultry, changes in the environment can regulate the secretion of hormones [34]. However, off-season breeding is a typical method of achieving economic growth by adjusting the rearing environment and feed management. GnRH is one of the key hormones involved in the regulation of seasonal reproduction in geese. Its secretion and synthesis are regulated by photoreceptors in the hypothalamus, its own reproductive function, and the endocrine system [35]. GnRH produced by the hypothalamus regulates LH and FSH production by the pituitary gland, and the continuous secretion of GnRH is regulated by other nerve centers, especially photoreceptors. In birds exposed to long days, the secretion of GnRH increases, causing an increase in plasma gonadotropin concentration and maintaining the ovarian production of somatotropin and follicle development. According to a study, the rhythmic secretion of GnRH activates the adenylate cyclase/cAMP/protein kinase system by binding to specific receptors on gonadotropin-secreting cells in the pituitary gland and induces the secretion of LH and FSH [36]. Changes in FSH and LH levels stimulate the secretion of E2 and P4 in follicular cells to regulate mating and other reproductive behaviors during the breeding period. Yang Haiming et al. [37] studied the expression of GnRH in Yangzhou geese under different light and temperature conditions and found that GnRH gene expression during the laying period was significantly higher than that during the rest period. The present study showed that GnRH and VIP were expressed in the hypothalamus, pituitary, and ovary of eastern Zhejiang white geese. GnRH gene expression in the three tissues in the off-season breeding group during the rest period was significantly lower than that in the control group during the peak laying period; specifically, GnRH expression in the hypothalamus showed a 10-fold difference, which is consistent with the previous findings, indicating that GnRH regulates the laying performance in eastern Zhejiang white geese. Moreover, the expression of FSH and LH genes in the pituitary tissue in the off-season breeding group during the rest period was significantly lower than that in the control group during the peak laying period, consistent with the higher serum FSH and LH levels during the peak period of egg production. The serum FSH and LH levels in the off-season breeding group were higher than those in the control group at various time points, indicating that off-season breeding in eastern Zhejiang white geese was also strictly regulated by the hypothalamic–pituitary–gonadal axis to maintain the peak laying period.

Estradiol (E2) is predominantly synthesized by follicular cells, ovarian tissue, and the placenta. Avian progesterone (P4) is primarily produced by ovarian granulosa cells and is regulated by follicle-stimulating hormone (FSH) and luteinizing hormone (LH). Gumulka et al. observed significantly elevated serum levels of E2 and P4 in Anding geese during the laying period compared with the rest period [38]. Zhao et al. [39] found that the serum E2 and P4 levels of female Wanxi white geese were low before the breeding season, gradually increased as the laying period began, reached a peak in the middle and late laying periods, and reached the lowest level at the end of the laying period. In the present study, the change trend of serum E2 and P4 levels in the off-season breeding group and the control group was similar, and the highest hormone levels were consistent with the peak of the laying period. The change trend was that the serum E2 and P4 levels peaked at the peak of the laying period, gradually decreased after the peak laying period, and gradually increased during the early laying period after recovery during the rest period. However, the serum E2 and P4 levels in the off-season breeding group were significantly higher than those in the control group at some time points; these differences were consistent with the finding that the off-season breeding group maintained a longer peak laying period.

T3 and T4 are important endocrine hormones that participate in nutrient absorption and metabolism, bone growth and development, and gonadal development. Pond et al. reported that thyroid gland removal in turkeys led to a failure of egg production in antenatal turkeys and the cessation of egg production in turkeys during the laying period [40]. Liu et al. used the estrogen-like effect of daidzein to promote an increase in thyroxine levels in Hy-Line Brown laying hens, which improved their laying performance [41]. Previous studies have shown that T3, T4, and GH are important regulators of animal reproduction [42,43]. T3 and T4 are essential for the initiation and maintenance of egg production and for molting and regulate reproduction via the hypothalamic–pituitary–gonadal axis in poultry [44]. They regulate reproductive function directly and indirectly by regulating PRL secretion. The study showed that the change trend of serum T3 and T4 levels in eastern Zhejiang white geese was similar between the off-season breeding group and the control group; the serum T3 level was generally higher in the peak laying period, whereas the serum T4 level was lower in the rest period and higher in the peak laying period. The results suggest that the changes in serum T3 and T4 levels are closely associated with the energy expenditure and maintenance of laying performance during the laying period.

Off-season breeding prolonged the duration of the peak laying period of eastern Zhejiang white geese, and the egg production in the off-season breeding group was 15.62% higher than that in the control group. The average economic benefit per goose in the off-season breeding group was 7.2% higher than that in the control group. Off-season breeding increased the serum ALB, TG, and P levels and AST activity and promoted the metabolism of proteins, lipids, and minerals, which is conducive to maintaining the laying performance during the peak laying period. The change trends of serum FSH, LH, E2, P4, PRL, T3, and T4 levels in the off-season breeding group were similar to those in the control group. The serum E2, FSH, LH, P4, and T3 levels were higher in the off-season breeding group than in the control group at some time points during the peak laying period, and the highest levels of serum PRL were consistent with the rest period. Reproduction in avian species is controlled by the hypothalamic–pituitary–gonadal axis (HPGA), and the HPGA is a comprehensive feedback system formed under central nervous regulation [45]. HPGA is regulated by multiple hormones, including GnRH from the hypothalamus, prolactin (PRL), follicle-stimulating hormone (FSH), and luteinizing hormone (LH) from the pituitary gland, as well as E2 and P4 from the gonads [46], with PRL regulating nesting behavior through the HPGA. During oviposition, the E2 from the ovary acts on the hypothalamus, enhancing the activity of dopamine and vasoactive intestinal peptide (VIP), which in turn promotes the synthesis and release of VIP. Through a feedback effect, this stimulates the secretion of PRL, thereby driving the nesting behavior [47]. In this study, the gene expression levels of GnRH in the hypothalamus, pituitary, and ovary and FSH and LH in the pituitary were significantly lower in the off-season breeding group than in the control group during the laying period, but the gene expression levels of VIP and PRL were significantly higher than those in the control group. It is not difficult to observe that the reduction in hormone content is conducive to the secretion of VIP and PRL, which increases the nesting behavior of poultry and promotes egg-laying.

## Figures and Tables

**Table 1:** Feed composition of Zhedong white geese during laying period.

Type of Raw Material	Concentrated Feed	Roughage Feed
Corn (%)	48	24
Barley (%)	30	15
Bran (%)	5	8.5
Soybean meal (%)	12	6
Rice chaff (%)	2	45
Premix feed (%)	3	1.5
Salt (%)	0.3	0.3

**Table 2:** Primer sequences of target genes.

Gene Name	Primer Sequence
β-actin	F: TGACGCAGATCATGTTTGAGA R: GCAGAGCGTAGCCCTCATAG
GnRH	F: CTGGGACCCTTGCTGTTTTG R: AGGGGACTTCCAACCATCAC
VIP	F: ACCAGTGTCTACAGCCATCTTTTG R: AGGTGGCTCAGCAGTTCATCTACA
FSH	F: GTGGTGCTCAGGATACTGCTTCA R: GTGCAGTTCAGTGCTATCAGTGTCA
LH	F: GACCCGGGAACCGGTGTA R: AGCAGCCACCGCTCGTAG
PRL	F: TGCTCAGGGTCGGGGTTTCA R: GCTTGGAGTCCTCATCGGCAAGTT

**Table 3:** Effect of breeding season on production performance of Zhedong white geese in a breeding cycle.

Project Types	Control	Out of Season Breeding Group
Egg Production (Pieces)	31,537	30,496
Average Egg Production (Pieces)	31.54	30.50
Fertilized Egg (Pieces)	24,942	24,600
Number of spawn (Number)	21,788	21,473
Stillborn Egg (Pieces)	2065	2053
Loss of Goose Seeding (Number)	1089	1074
Hatching Rate of Eggs (%)	79.08	80.66
Hatching Rate of Fertilized Eggs (%)	69.09	70.04
Still Birth Rate (%)	87.35	87.28
Total Sales Amount of Goose Seeding (%)	8.28	8.35
Total Sales Amount of Goose Seeding (RMB)	491,170	526,702
The Economic Benefic of The Average Goose (RMB)	491.17	526.70

## Data Availability

The datasets used and/or analyzed during the current study are available from the corresponding author on reasonable request.

## Abbreviations

ALB (albumin), AST (glutamic oxalacetic transaminase), BUN (blood urea nitrogen), CREA (creatinine), E2 (estradiol), FSH (follicle-stimulating hormone), GnRH (gonadotropin releasing hormone), PRL (prolactin), P4 (progesterone), TG (triglyceride), TC (total cholesterol), T3 (triiodothyronine), T4 (Tetraiodothyronine), LH (luteinizing hormone), VIP (vasoactine intestinal peptide).
